# Promotion of ultra-processed foods in Brazil: combined use of claims and promotional features on packaging

**DOI:** 10.11606/s1518-8787.2023057004410

**Published:** 2023-07-18

**Authors:** Giovanna Calixto Andrade, Laís Amaral Mais, Camila Zancheta Ricardo, Ana Clara Duran, Ana Paula Bortoletto Martins

**Affiliations:** I Universidade de São Paulo Faculdade de Medicina Departamento de Medicina Preventiva São Paulo SP Brasil Universidade de São Paulo. Faculdade de Medicina. Departamento de Medicina Preventiva. São Paulo, SP, Brasil; II Universidade de São Paulo Núcleo de Pesquisas Epidemiológicas em Nutrição e Saúde São Paulo SP Brasil Universidade de São Paulo. Núcleo de Pesquisas Epidemiológicas em Nutrição e Saúde. São Paulo, SP, Brasil; III Instituto Brasileiro de Defesa do Consumidor São Paulo SP Brasil Instituto Brasileiro de Defesa do Consumidor. São Paulo, SP, Brasil; IV Universidad de Chile. Facultad de Mecicina Escola de Salud Pública Santiago Chile Universidad de Chile. Facultad de Mecicina. Escola de Salud Pública. Santiago, Chile; V Universidade Estadual de Campinas Núcleo de Estudos e Pesquisas em Alimentação Campinas SP Brasil Universidade Estadual de Campinas. Núcleo de Estudos e Pesquisas em Alimentação. Campinas, SP, Brasil

**Keywords:** Ultra-Processed Food, Marketing, Promotional Features, Claims, Food Labels, Packaging

## Abstract

**OBJECTIVE:**

To assess the availability of different promotional strategies applied for UPF sales in Brazilian food retailers.

**METHODS:**

Information available on food packaging was gathered from all packaged products sold in the five largest food retail chains in Brazil in 2017. UPF were identified using the NOVA food classification system. From this sample, data related to promotional characteristics, nutrition claims and health claims were collected and coded using the INFORMAS methodology. Additional claims referring to the Brazilian Dietary Guidelines were also collected.

**RESULTS:**

This study evaluated the packaging of 2,238 UPF, of which 59.8% presented at least one promotional strategy. Almost one third denoted a simultaneous use of different promotional strategies in the same packaging. Nutrition claims were the most commonly found promotional strategy, followed by health claims and the use of characters. The food subgroups comprising the highest prevalence of promotional strategies on their labels were: noncaloric sweeteners (100.0%), breakfast cereals and granola bars (96.2%), juices, nectars and fruit-flavoured drinks (92.9%), other unsweetened beverages (92.9%), and other sweetened beverages (92.6%).

**CONCLUSIONS:**

Considering the poor nutritional quality of UPF, the widespread presence of promotional features on their packaging highlights the need for marketing restrictions on this kind of product.

## INTRODUCTION

Industrialization, urbanization, economic development, and market globalization are associated with changes in the diets and lifestyles of the population, fomenting the increasing prevalence of overweight and obesity^1^. The transition from traditional culinary to the consumption of highly processed foods is one of the main alterations perceived in the food habits of the population^2^. In Brazil, national surveys periodically conducted showed that overweight prevalence increases^3^ concomitantly with the acquisition of ultra-processed foods (UPF)^4^. Moreover, studies brought to light a positive association between UPF consumption and the development of chronic diseases such as obesity, diabetes, cardiovascular diseases, and some types of cancer, among others^5^.

Several mechanisms can explain the association between UPF consumption and both the decline in diet quality and weight gain. High energy density, sugar, fat, and sodium content^10,11^, large portions, and the high palatability of these products are some of the characteristics that stimulate excessive caloric intake^11^. Additionally, marketing strategies applied for food sales create an environment that promotes excessive food consumption and contributes to the obesity epidemic^12^. In Brazil, a study discussing the accelerated growth of UPF consumption showed that this phenomenon was possibly driven by the combination of food palatability, advertising, and other aspects^4^.

Food packaging stands out as one of the main marketing strategies to promote UPF. The packaging is responsible for conveying the attributes of the product to general consumers^15^, representing a chief communication tool at the moment of purchase, which is essential to attract consumers’ attention and influence their decision-making^16,17^. Health and nutrition claims, for example, lead the consumers to conclude that the product is healthy, influencing their purchase^18^. Promotional features on food packaging, on the other hand, add value to the product by aggregate sales or the use of characters or celebrities, influencing the decision-making process^19,20^. Children and adolescents are even more susceptible to food marketing^21^ since their choices are influenced by “fun” elements, such as characters (brand or licensed) and gifts in the package^19^.

However, studies evaluating food marketing strategies in Brazil, as well as in other Latin American countries, are more focused on television advertising, with few studies reporting marketing on food labels^22^. Studies evaluating food packaging in Brazil are more focused on a specific nutrient or ingredients^23,24^, or exclusively evaluate the use of nutrition and health claims^25,26^.

Identifying the main promotional strategies present on UPF packaging marketed in Brazil may represent one of the chief approaches used to develop public policies focusing on diet quality improvement and, consequently, supporting the struggle against obesity. Thus, this study aimed to assess the availability of different promotional strategies implemented on UPF packages sold in Brazilian supermarkets, compare the prevalence of these strategies in other food groups’ packaging, and evaluate the co-occurrence of different types of promotional features on the packaging.

## METHODS

This is a cross-sectional study based on the data gathered from labels of packaged foods and beverages sold in the five largest supermarket chains in Brazil. This information was collected from April to July 2017.

Supermarkets were selected as the source of data collection because they make all types of foods and food brands available to the population, which are responsible for a large share of the energy consumed by Brazilians^27^. Euromonitor International’s annual sales data was used to identify the largest food retailers in the country, which account for close to 70% of the edible grocery banner sales in Brazil^28^. São Paulo was chosen as the primary study area because it is the largest city in Brazil. Since one of the largest food retailers is located in Northeastern Brazil, Salvador was selected as it is the largest city in the region.

Data on the location of every store of these five retail chains in São Paulo and Salvador were gathered from each company’s website, and the addresses were geocoded. To select the stores, a 1 km buffer was estimated around each store of the selected supermarket chains, then the *per capita* household income average information available from the Brazilian Demographic Census 2010 was used^29^. Subsequently, the addresses of all stores were distributed according to the *per capita* household income. Stores of the first and last tertiles were selected to ensure socioeconomic representativeness in the sample, prioritizing those with larger areas. Formal permission was obtained from all supermarket chains included in this study. All packaged foods and beverages found in each store were included in the sample. Information from each side of all packages were photographed by previously trained fieldworkers, according to the methods proposed by Kanter et al.^30^ Afterwards, mandatory information from packaged foods, such as brand, origin, list of ingredients, and nutrition facts panel, was entered by trained nutritionists into the online platform RedCap, using a form adapted for Brazilian markets based on the form developed by the University of North Carolina at Chapel Hill (UNC) from the United States of America (USA) and by the *Instituto de Nutrición y Tecnología de los Alimentos* (INTA) from Chile. Duplicated items and products available in more than one package size were excluded, keeping only one size for each item. Products without nutrition information and with multiple items were also excluded. In total, information was collected from 11,434 products.

Among these products, a representative subsample consisting of 3,491 products (30%) was randomly selected. This sample was drawn from each of the 128 categories of food primarily used in data entering. No statistical differences were found in food composition when this random sample was compared with the universe of photographed food packages. Among the subsample products, data related to nutrition and/or health claims, and promotional characteristics were collected and coded using the International Network for Food and Obesity/Non-communicable Diseases (NCDs) Research, Monitoring and Action Support (INFORMAS) methodology^31^. This methodology was developed by INFORMAS to monitor different aspects of food packaging. The proposed taxonomy has a step-wise approach that was developed for independently assessing the nature and extent of health-related food labeling in different countries and over time. The INFORMAS protocol divided the food labeling components into three main groups: Nutrient declaration (including information such as the nutrition label), Nutrition and health claims, and Promotional characters and premium offers. In this study, the last two components were evaluated.

Nutrition and health claims are used on food packaging by the food industry to inform consumers of a health benefit that a product may have. Health claims are the ones related to general beneficial health, allergies/intolerance, vegetarian/vegan content, natural/pure products, products without additives, pesticides, hormones, nutrient function, and/or risk/protection of disease. Nutrition claims include products declaring the content or comparing nutrients with health-related ingredients (such as fruits, nuts, and whole grains). Promotional characters and premium offers include information on the presence of characters (branded, licensed, and movies characters or celebrities), sports events or athletes (famous or amateur), and awards (game download, contests, promotions such as “buy 2 and get 3” and “extra percentage of the product,” limited edition, social charity, and collectible item)^31^.

Claims referring to the Brazilian Dietary Guidelines (BDG) were also collected. This document is the official recommendation on healthy diets of the Ministry of Health and is the first guideline to present the broad and innovative approach for food classification based on industrial food processing degree and purpose (NOVA classification). The main recommendation is a diet based on fresh (*in natura*) and minimally processed foods, thus avoiding the consumption of UPF. The document also endorses that meals should be prepared, consumed, and shared with family and/or friends, encouraging commensality^32^. Therefore, messages covering the degree of industrial food processing and commensality were measured using the following items: 1) messages referring to the level of industrial food processing or the amount of ingredients; 2) content stating if the food is fresh and/or straight from the farm; 3) information referring to commensality (as eating together or sharing food); and, 4) messages directly mentioning the BDG. Supplementary Material 1^a^ shows more details on the promotional strategies evaluated in this study.

Information regarding food promotional features and claims was entered twice. Moreover, intra and interrater reliability analyses were performed and evaluated using Cohen’s kappa coefficient. According to the criteria suggested in the literature, the agreement level was interpreted as follows: 0.01 to 0.20 – slight; 0.21 to 0.40 – fair; 0.41 to 0.60 – moderate; 0.61 to 0.80 – substantial; and 0.81 to 1.00 – almost perfect or perfect agreement^33^. The collected data was considered reliable.

All products were categorized according to the NOVA food classification system, which divides foods and beverages into four groups: 1) unprocessed or minimally processed foods (items obtained directly from plants or animals without processing or with minimal alteration); 2) processed culinary ingredients (substances extracted from natural foods or from nature and consumed for culinary preparation); 3) processed foods (manufactured products prepared essentially by adding processed culinary ingredients—such as salt, sugar, and oil—to unprocessed or minimally processed foods to extend shelf life and improve palatability); and 4) ultra-processed foods and beverages (made mainly or solely of industrial ingredients, their production process involves complex manufacturing techniques used exclusively by the industry)^34^. Supplementary Material 2^b^ shows food groups and the definition of the NOVA classification.

UPF were further divided according to their resemblance into 18 subgroups: breakfast cereals and granola bars, bakery products, convenience foods, dairy products, ultra-processed cheeses, salty snacks, crackers, cookies, canned vegetables, margarine, sauces and dressings, candies and desserts, noncaloric sweeteners, ultra-processed meats, juices, nectars and fruit-flavored drinks, sodas, other sweetened beverages, other unsweetened beverages. Following the definition of the NOVA classification, all items containing aesthetic food additives were considered UPF.

To compare the use of promotional strategies in the packaging of products categorized according to the NOVA classification, first we estimated the prevalence (%) and respective 95% confidence intervals (95%CI) of promotional strategies and prevalence of products with more than one promotional strategy on the packaging of unprocessed or minimally processed foods, processed culinary ingredients, processed foods, and UPF. In sequence we estimated the prevalence of products without a promotional strategy, with one type of promotional strategy, and with two or more types of promotional strategies among UPF and other food products.

To assess the availability of different promotional strategies implemented on UPF packages sold in Brazilian supermarkets, we calculated the prevalence (%) and respective 95%CI of different types of promotional strategies among UPF and for each of the 18 subgroups of UPF.

Cluster analyses were used to evaluate the co-occurrence of different types of promotional strategies among UPF. The clustering pattern was studied using a comparison between observed prevalence (OP) and expected prevalence (EP). Clustering occurs when the observed prevalence exceeds the expected prevalence of the combination. The expected prevalence for each combination was acquired by multiplying the probabilities of each promotional strategy. A total of 56 possible combinations of the six types of promotional strategies were studied. Clustering was defined when a combination was more prevalent than expected, based on the prevalence of each isolated risk, i.e., a combination in which the ratio OP/EP was greater than one.

All analyses were performed using the program Stata 14.0 (StataCorp, Texas, USA).

## RESULTS

From the 3,491 products analyzed, 797 (22.8%) were classified as unprocessed or minimally processed foods, 100 (2.9%) as processed culinary ingredients, 356 (10.2%) as processed foods, and 2,238 (64.1%) as UPF. The total prevalence of promotional features in the food groups was 52.1% (95%CI 48.6–55.5), 56.0% (95%CI 46.0–65.5), 60.7% (95%CI 55.5–65.6), and 59.8% (95%CI 57.8–61.8), respectively. Around 28.1% of the evaluated products presented more than one type of promotional strategy on the packaging. This prevalence was higher among UPF (29.6%), and lower among unprocessed or minimally processed foods (24.3%) and processed culinary ingredients (19.0%) (Table 1).

**Table 1:** Distribution (%) of the presence of promotional strategies on food packages, according to the categories of NOVA classification. Brazil, 2017.

Characteristic	Total sample	Products without promotional strategy		Products with promotional strategies		Products with two or more promotional strategies	
			
	n (%)	%	95%CI	%	95%CI	%	95%CI
Unprocessed or minimally processed foods	797 (22.8)	47.9	44.4–51.4	52.1	48.6–55.5	24.3	21.5–27.5
Processed culinary ingredients	100 (5.9)	44.0	34.5–54.0	56.0	46.0–65.5	19.0	12.4–28.1
Processed foods	356 (10.2)	39.3	34.4–44.5	60.7	55.5–65.6	28.4	23.9–33.3
Ultra-processed foods	2,238 (64.1)	40.2	38.1–42.2	59.8	57.8–61.8	29.8	27.9–31.8
**Total**	**3,491 (100)**	**42.0**	**40.3–43.6**	**58.0**	**56.4–59.7**	**28.1**	**26.6–29.6**

95%CI: 95% confidence interval.

The figure compares the presence of promotional strategies in UPF with other food products. Among UPF, a higher prevalence of products with promotional strategies was found on the packaging (59.8%) when compared with the other food products (54.8%). UPF also showed higher use of two or more different promotional strategies in the same packaging (29.8%) when compared with the other food products (25.1%).

**Figure:**
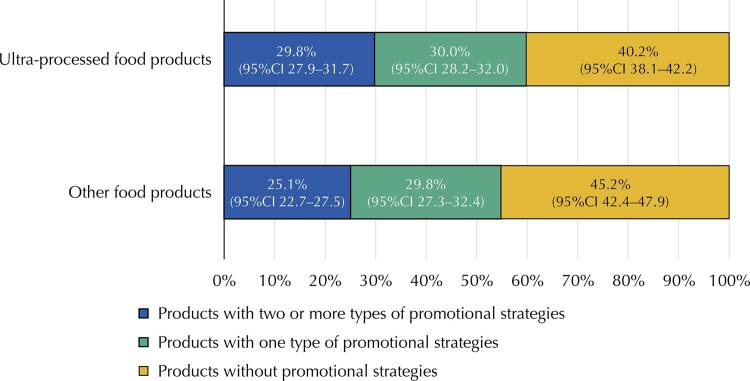
Comparison of the prevalence of promotional strategies used on the packaging of ultra-processed foods and other food products marketed in Brazilian supermarket chains, 2017.

95%CI: 95% confidence interval.

The most common type of promotional strategy observed on the packaging of the analyzed products was health claims (33.3%), followed by nutrition claims (32.1%), use of characters (19.8%), claims referring to the BDG (5.0%), awards (2.9%), and use of athletes or sports events (0.8%). Among unprocessed or minimally processed foods and processed foods, the most prevalent promotional strategy was the use of health claims (34.5% and 35.1.5, respectively). Among processed culinary ingredients, health claims (24.0%) and the use of characters (23.0%) were the most common promotional strategy. The most common promotional strategies for UPF were the use of nutrition claims (36.1%) and health claims (32.9%) (Table 2).

**Table 2:** Prevalence of different types of promotional strategies in the Brazilian food supply by degree of industrial food processing, 2017.

Characteristics	Characters		Athletes/sports events		Awards		Claims referring to the BDG		Nutrition claims		Health claims	
					
	%	95%CI	%	95%CI	%	95%CI	%	95%CI	%	95%CI	%	95%CI
Unprocessed or minimally processed foods	17.6	15.1–20.4	1.4	0.8–2.5	1.5	0.9–2.6	6.0	4.6–7.9	24.1	21.2–27.2	34.5	31.3–37.9
Processed culinary ingredients	23.0	15.7–32.3	0	-	5.0	2.1–11.5	6.0	2.7–1.3	19.0	12.4–27.9	24.0	16.6–33.4
Processed foods	22.5	18.4–27.1	0	-	3.6	2.1–6.1	5.1	3.2–7.9	28.4	23.9–33.3	35.1	30.3–40.2
Ultra-processed foods	20.1	18.4–21.8	0.8	0.5–1.3	3.2	2.5–4.0	4.6	3.8–5.5	36.1	34.1–38.1	32.9	31.0–34.9
**Total**	**19.8**	**18.5–21.2**	**0.8**	**0.6–1.2**	**2.9**	**2.4–3.5**	**5.0**	**4.3–5.8**	**32.1**	**30.5–33.6**	**33.3**	**31.7–34.8**

BDG: Brazilian Dietary Guidelines; 95%CI: 95% confidence interval.

The most frequent subgroups of UPF were candies and desserts (n = 432), followed by convenience foods (n = 253), sauces and dressings (n = 205), ultra-processed meats (n = 193), cookies (n = 171), bakery products (n = 151) and dairy products (n = 150). The prevalence of promotional strategies according to UPF subgroups was noticeably extensive, ranging from 14.3% to 100%. Noncaloric sweeteners, breakfast cereals and granola bars, juices, nectars and fruit-flavored drinks, other unsweetened beverages, and other sweetened beverages were the subgroups presenting the highest prevalence of products with promotional strategies and containing more than one type of promotional strategy on the packaging (Table 3).

**Table 3:** Prevalence of promotional strategies used on the packaging of ultra-processed food subgroups sold in Brazilian supermarkets, 2017.

Characteristic	Total sample	Products with promotional strategy		Products with two or more promotional strategies	
		
	n	%	95%CI	%	95%CI
Breakfast cereals and granola bars	79	96.2	88.8–98.8	86.1	76.5–92.1
Bakery products	151	64.2	56.3–71.5	35.8	28.5–43.7
Convenience foods	253	56.5	50.3–62.5	24.9	19.9–30.6
Dairy products	150	75.3	67.8–81.6	29.3	22.6–37.1
Ultra–processed cheeses	115	57.4	48.2–66.1	18.3	12.2–26.4
Salty snacks	100	56.0	46.1–65.4	22.0	14.9–31.2
Crackers	42	57.1	41.8–71.2	19.0	9.7–33.9
Cookies	171	59.6	52.1–66.8	35.1	28.3–42.6
Canned vegetables	56	14.3	7.3–26.2	5.4	1.7–15.5
Margarine	32	84.4	67.2–93.4	50.0	33.1–66.9
Sauces and dressings	205	42.0	35.4–48.8	14.6	10.4–20.2
Candies and desserts	432	55.8	51.1–60.4	25.5	21.6–29.8
Noncaloric sweeteners	15	100.0	-	60.0	34.0–81.4
Ultra-processed meats	193	37.8	31.2–44.9	13.5	9.3–19.1
Juices, nectars, and fruit-flavored drinks	126	92.9	86.8–96.3	58.7	49.9–67.0
Sodas	35	51.4	35.1–67.5	17.1	7.8–33.6
Other sweetened beverages	41	92.7	79.4–97.7	65.9	50.1–78.8
Other unsweetened beverages	42	92.9	79.8–97.7	61.9	46.4–75.3

95%CI: 95% confidence interval.

Table 4 shows the presence of different promotional strategies in UPF subgroups. Characters appeared especially on other unsweetened beverages (35.7%) and ultra-processed cheeses (34.8%) packages, while athletes/sporting events had a lower appearance, but appeared especially on sodas (8.6%) and margarine (6.3%) packages. Crackers (9.5%) and juices, nectars, and fruit-flavored drinks (7.1%) showed the highest use of awards, and other sweetened beverages (16.7%) and juices, nectars, and fruit-flavored drinks (15.1%) showed the highest use of claims referring to the BDG. Finally, nutrition and health claims appeared in many packages, mainly on breakfast cereals and granola bars (93.7% and 78.5%, respectively), sugar and low-calorie table and baking sweeteners (86.7% and 73.3%, respectively), and other sweetened beverages (75.6%, in the case of health claims).

**Table 4:** Prevalence of different types of promotional strategies used on the packaging of ultra-processed food subgroups sold in Brazilian supermarkets, 2017.

Characteristic	Characters		Athletes/ sporting events		Awards		Claims referring to the BDG		Nutrition claims		Health claims	
					
	%	95%CI	%	95%CI	%	95%CI	%	95%CI	%	95%CI	%	95%CI
Breakfast cereals and granola bars	24.1	15.9–34.7	3.8	1.2–11.2	3.8	1.2–11.2	11.4	6.0–20.5	93.7	85.6–97.4	78.5	68.0–86.2
Bakery products	17.9	12.5–24.8	0.0	-	2.0	0.6–6.0	3.3	1.4–7.7	41.7	34.1–49.8	40.4	32.8–48.4
Convenience foods	22.1	17.4–27.7	0.0	-	2.0	0.8–4.7	6.3	3.9–10.1	30.8	25.4–36.8	30.8	25.4–36.8
Dairy products	28.7	22.0–36.4	0.0	-	1.3	0.3–5.2	1.3	0.3–5.2	36.0	28.7–44.0	44.0	36.2–52.1
Ultra-processed cheeses	34.8	26.6–44.0	1.7	0.4–6.7	0.0	-	2.6	0.8–7.8	29.6	21.9–38.6	10.4	6.0–17.5
Salty snacks	18.0	11.6–26.8	3.0	1.0–8.9	5.0	2.1–11.5	7.0	3.4–14.0	26.0	18.3–35.5	26.0	18.3–35.5
Crackers	9.5	3.6–23.0	0.0	-	9.5	3.6–23.0	0.0	-	38.1	24.7–53.6	23.8	13.2–39.1
Cookies	14.6	10.1–20.8	0.6	0.1–4.1	6.4	3.6–11.3	4.1	2.0–8.4	42.7	35.5–50.2	33.3	26.7–40.8
Canned vegetables	5.4	1.7–15.5	0.0	-	0.0	-	0.0	-	5.4	1.7–15.5	8.9	3.7–19.9
Margarine	25.0	12.9–42.9	6.3	1.5–22.2	0.0	-	6.3	1.5–22.2	62.5	44.6–77.5	46.9	30.3–64.1
Sauces and dressings	11.2	7.6–16.3	0.0	-	3.9	2.0–7.6	2.9	1.3–6.4	20.5	15.5–26.6	20.0	15.1–26.1
Candies and desserts	23.6	19.8–27.9	0.5	0.1–1.8	3.5	2.1–5.7	3.0	1.8–5.1	29.2	25.1–33.6	27.3	23.3–31.7
Sugar and low-calorie table and baking sweeteners	0.0	-	0.0	-	0.0	-	0.0	-	86.7	58.2–96.8	73.3	45.7–90.0
Ultra-processed meats	21.8	16.5–28.2	0.0	-	1.0	0.3–4.1	1.6	0.5–4.7	12.4	8.5–17.9	17.1	12.4–23.1
Juices, nectars, and fruit-flavored drinks	11.1	6.7–17.9	0.0	-	7.1	3.7–13.2	15.1	9.8–22.5	74.6	66.3–81.5	64.3	55.5–72.2
Sodas	5.7	1.4–20.5	8.6	2.7–23.8	5.7	1.4–20.5	5.7	1.4–20.5	42.9	27.5–59.7	8.6	2.7–23.8
Other sweetened beverages	19.5	10.0–34.6	4.9	1.2–17.8	4.9	1.2–17.8	2.4	0.3–15.7	75.6	60.1–86.5	75.6	60.1–86.5
Other unsweetened beverages	35.7	22.7–51.3	0.0	-	0.0	-	16.7	8.1–31.3	52.4	37.3–67.0	64.3	48.7–77.3

BDG: Brazilian Dietary Guidelines; 95%CI: 95% confidence interval.

Table 5 shows the prevalence of promotional strategies co-occurrence, with the OP and the EP and the OP/EP for all possible combinations of the six promotional strategies in UPF. OP/EP ratio above one was observed in 19 out of 63 possibilities, corresponding to a clustering of promotional strategies. The highest OP/EP ratios were found for the combination of nutritional and health claims, characters, athletes/sporting events and awards (OP/EP 76.9), the combination of nutritional and health claims, athletes/sporting events and awards (OP/EP 19.3) and the combination of nutritional and health claims, athletes/sporting events and BDG claims (OP/EP 13.2) (Table 5).

**Table 5:** Clustering patterns of promotional strategies presented on the packaging of ultra-processed foods subgroups sold in Brazilian supermarkets, 2017.

Number of promotional strategies	Characters	Athletes or sports events	Awards	BDG claims	Nutrition claim	Health claim	Expected prevalence (%)	Observed prevalence (%)	Observed/ Expected prevalence
6	+	+	+	+	+	+	0.00	0.00	0.00
5	-	+	+	+	+	+	0.00	0.00	0.00
	+	-	+	+	+	+	0.00	0.00	0.00
	+	+	-	+	+	+	0.00	0.00	0.00
	+	+	+	-	+	+	0.00	0.04	76.92
	+	+	+	+	-	+	0.00	0.00	0.00
	+	+	+	+	+	-	0.00	0.00	0.00
4	-	-	+	+	+	+	0.01	0.00	0.00
	-	+	-	+	+	+	0.00	0.04	13.25
	-	+	+	-	+	+	0.00	0.04	19.31
	-	+	+	+	-	+	0.00	0.00	0.00
	-	+	+	+	+	-	0.00	0.00	0.00
	+	-	-	+	+	+	0.10	0.49	4.71
	+	-	+	-	+	+	0.07	0.18	2.49
	+	-	+	+	-	+	0.01	0.00	0.00
	+	-	+	+	+	-	0.01	0.00	0.00
	+	+	-	-	+	+	0.02	0.04	2.52
	+	+	-	+	-	+	0.00	0.00	0.00
	+	+	-	+	+	-	0.00	0.00	0.00
	+	+	+	-	-	+	0.00	0.00	0.00
	+	+	+	-	+	-	0.00	0.00	0.00
	+	+	+	+	-	-	0.00	0.00	0.00
3	-	-	-	+	+	+	0.42	1.56	3.76
	-	-	+	-	+	+	0.29	0.49	1.72
	-	-	+	+	-	+	0.02	0.00	0.00
	-	-	+	+	+	-	0.03	0.00	0.00
	-	+	-	-	+	+	0.07	0.22	3.16
	-	+	-	+	-	+	0.01	0.00	0.00
	-	+	-	+	+	-	0.01	0.00	0.00
	-	+	+	-	-	+	0.00	0.00	0.00
	-	+	+	-	+	-	0.00	0.04	9.48
	-	+	+	+	-	-	0.00	0.00	0.00
	+	-	-	-	+	+	2.19	3.22	1.47
	+	-	-	+	-	+	0.18	0.13	0.73
	+	-	-	+	+	-	0.21	0.09	0.42
	+	-	+	-	-	+	0.13	0.00	0.00
	+	-	+	-	+	-	0.15	0.40	2.76
	+	-	+	+	-	-	0.01	0.00	0.00
	+	+	-	-	-	+	0.03	0.04	1.42
	+	+	-	-	+	-	0.04	0.04	1.24
	+	+	-	+	-	-	0.00	0.00	0.00
	+	+	+	-	-	-	0.00	0.00	0.00
2	-	-	-	-	+	+	8.71	14.34	1.65
	-	-	-	+	-	+	0.74	1.07	1.46
	-	-	-	+	+	-	0.85	0.13	0.16
	-	-	+	-	-	+	0.51	0.13	0.27
	-	-	+	-	+	-	0.58	0.31	0.54
	-	-	+	+	-	-	0.05	0.00	0.00
	-	+	-	-	-	+	0.13	0.09	0.71
	-	+	-	-	+	-	0.14	0.09	0.62
	-	+	-	+	-	-	0.01	0.00	0.00
	-	+	+	-	-	-	0.01	0.00	0.00
	+	-	-	-	-	+	3.87	2.37	0.61
	+	-	-	-	+	-	4.45	3.49	0.78
	+	-	-	+	-	-	0.38	0.27	0.71
	+	-	+	-	-	-	0.26	0.36	1.38
	+	+	-	-	-	-	0.06	0.04	0.70
1	-	-	-	-	-	+	15.42	8.40	0.54
	-	-	-	-	+	-	17.74	10.81	0.61
	-	-	-	+	-	-	1.50	0.76	0.51
	-	-	+	-	-	-	1.03	1.16	1.13
	-	+	-	-	-	-	0.25	0.04	0.18
	+	-	-	-	-	-	7.88	8.85	1.12

BDG: Brazilian Dietary Guidelines.

## DISCUSSION

About 60% of the UPF available in the Brazilian market presented at least one promotional strategy on their packaging. Nutrition claims were the most common strategy on UPF packaging, followed by health claims, use of characters, claims related to BDG, awards, and the presence of athletes or sports events. These results are concerning since promotional strategies have a significant influence on the moment of purchase and may induce consumers to choose unhealthy products^18^.

Health and nutrition claims, for example, can influence consumers to erroneously conclude that certain food products are healthy, inducing food purchases and possibly leading to the excessive consumption of these foods^18^. These claims are regulated by the Brazilian legislations RDC nº 360 and RDC nº 18, which allows the use of health and nutrition claims if there is scientific proof of the functional properties or the health statement. If the packaging contains a declaration of a nutritional property or refers to a nutrient content, the amount of that nutrient must be declared, but the legislation does not require a minimum content of the nutrient for the use of the claims. Also, Brazilian legislation does not consider the nutritional quality of the product as a whole^35,36^. A study conducted in Brazil indicates that part of the commercialized products with nutrition and health claims presented poorer nutritional quality^25^. In this sense, the high prevalence of health and nutrition claims found in UPF highlights the need to review the legislation.

Promotional features on food packaging, on the other hand, add value to the product by aggregate sales or the use of characters or celebrities, influencing on the decision-making process. Children and adolescents are particularly influenced by promotional strategies, such as characters, awards, and sports events or athletes, which are frequently employed in unhealthy food packaging to attract youth^19,20,37^. In this study, characters were observed in all UPF subgroups except for noncaloric sweeteners. The use of athletes or sports events, which not only attracts young people^39^ but associates the product with health messages^42^, was more frequent among foods characterized by high sugar content, such as sodas, other sweetened beverages, breakfast cereals, and granola bars. Moreover, awards such as game downloads, contests, promotions, and collectible items were more frequent among crackers, cookies, sodas, and salty snacks, which are items frequently consumed by children and teenagers in Brazil^43^.

The use of promotional strategies in the packaging of healthy foods can encourage the purchase of the product; however, studies demonstrate that its use has a greater influence on the consumption of unhealthy foods when compared with healthy foods among children^37,44,45^. When presented with a choice between two healthy foods, one with a known character on the packaging and the other without, children tend to choose the item with character^44^ and report it as tastier^37^. However, this effect is stronger when used in the packaging of unhealthy foods^37,44^. Additionally, if a child needs to decide between a fruit/vegetable with a branded character on its label and an energy-dense food with the same character, they tend to select the energy-dense food^44,45^.

The high palatability, appetizing, and attractiveness of UPF^10,46^ may explain why promotional strategies between these products have a greater influence on the food choices of consumers. Researchers suggest that food marketing attracts youth, while highly palatable ingredients positively reinforce its consumption^47,48^. The visual attraction of food may be another factor that justifies the preference for UPF over other foods, as highlighted by different marketing strategies. A study using food images showed that UPF provokes an appetitive motivation, often leading to extremely arousing and pleasant reactions that have been associated with food craving ratings and addictive behaviors to other substances^49^.

The last measured promotional strategy was the presence of claims referring to the BDG. This was the fourth most popular promotional feature observed on UPF packaging, with “natural” or “*in natura*” as the most frequent citations. Although this type of statement was already present on packaging before the BDG, it is concerning that the food industry has appropriated and distorted an official health concept described by the Brazilian Ministry of Health to promote the sale of unhealthy food products, which conflicts with the BDG recommendations.

The prevalence of promotional strategies on food packaging was remarkable in all food groups evaluated in this study, and its elevated use in UPF should be highlighted due to their poor nutritional quality^10,11^, their association with the development of diseases^5^, and greater marketing influence on the labeling of low nutritional quality foods^37,44,45^.

Almost one-third of UPF sold in the country presented the simultaneous use of different promotional strategies in the same packaging. This can reach out to different audiences (e.g., adults and children) and augment the products’ value, increasing the power of the advertised message. The combination of nutrition and health claims could reinforce the erroneous idea that determined food is healthy since they are two different misleading concepts in products that are proven to be unhealthy. In contrast, combining strategies such as awards with nutrition and health claims attract the consumer by fomenting the idea of healthy food and providing the “advantage” of receiving an associated award. This combination could influence different audiences, attracting young people because of the award and adults because of the idea of a healthy product. The combined use of promotional strategies in UPF packaging may increase the persuasive power of the message; however, further studies are needed to understand the effects of the combined use of promotional strategies on food packaging.

The World Health Organization (WHO) recognizes advertising on low nutritional quality foods as one of the factors associated with the global obesity epidemic and recommends its regulation, especially among youth, which is the most susceptible age group to advertising^50^. Some countries have advanced when dealing with the regulation of food marketing targeted at children and adolescents by limiting fast food and television advertisements^51^, but only Chile has advanced on food marketing and food labeling in the same legislation. In addition to implementing front-of-package warning labels on products with high amounts of critical nutrients, Chilean Law no. 20,606, published in June 2016, also regulates advertising to children in UFP packaging, banning the use of characters and gifts^52^.

In Brazil, advertising content aimed at youth is regulated by the *Conselho Nacional dos Direitos da Criança e do Adolescente* (CONANDA). Although the council defines the use of celebrities, characters, and distribution of prizes or gifts as abusive marketing practices (Resolution no. 163/2014)^53^, the National Congress decreed in April 2014 that CONANDA could not legislate on advertising^54^.

The low regulation in the advertising of UPF in Brazil is probably a reflection of the great influence of the food industry in political decision-making. This can be observed in the recent process of reviewing the regulation of nutrition labeling for packaged foods in Brazil. In 2020, the Resolution of the Collegiate Board (*Resolução da Diretoria Colegiada* - RDC) no. 429/2020 and the Normative Instruction (*Instrução Normativa* - IN) no. 75/2020 were published^55^. Although the regulation made front-of-package nutrition labeling mandatory, the model adopted in Brazil utilized lower cut-off points than other Latin American countries and did not prohibit the use of nutrition and health claims on products high in critical nutrients^55,56^.

We found a lower prevalence of claims and other packaging promotional strategies in UPFs sold in Brazil than in studies conducted in high-income countries^40,57,58^. In Australia, for example, almost all UPF have at least one marketing strategy in the packaging of foods and beverages^59^. These differences can be explained by divergences between consumers in developed and developing countries. Low-income individuals are likely to be more price sensitive than those living in high-income countries^60^, which may shift food companies’ strategies to offering cheaper products in low- and middle-income countries, such as Brazil. Additionally, companies may choose to invest in other sales strategies besides food labeling. However, more studies are needed to trace the consumer profile and other marketing strategies used by the food industry.

This study has some limitations. The outcomes are limited to describing only the promotional features present on food packaging commercialized in Brazil, disregarding the presence of other marketing strategies present on packaging (e.g. design and colors) and marketing strategies at supermarkets (such as shelves layout and occupation, sale boxes, and sale islands). Additionally, unpackaged foods, such as fruits and vegetables, were not included, and the prevalence of promotional features in unprocessed and minimally processed foods is limited to packaged foods. Since unpackaged foods were not considered, the prevalence of minimally processed foods with promotional strategies is likely overestimated; therefore, it is plausible to state that the prevalence of advertisements on UPF packaging is much higher than on *in natura* foods, as many foods in this group are not factory-packaged.

Despite its limitations, this study stands out due to its large sample size. This is the first study evaluating the promotional features of UPF packaging in Brazil using the INFORMAS protocol, which was developed to standardize the classification of different health-related labeling components and promotional features present on food packaging in different countries. By using this protocol, it is possible to compare labels from different countries and enable continuous monitoring of promotional strategies^31^.

## IMPLICATIONS FOR RESEARCH AND PRACTICE

Considering the influence of food packaging advertising on consumers’ food choices, specifically those related to high energy-density and low nutritional quality foods, expanding and improving label regulation of foods and beverages marketed in Brazil is necessary. Legislation regulating nutrition or health claims should limit their use in UPF. Furthermore, promoting healthy eating habits and prohibiting the use of characters, celebrities, athletes, sports events, and awards on UPF packaging is essential, especially due to its already proven influence on children’s and adolescents’ eating choices.

## Footnotes

a Available from: https://drive.google.com/file/d/1vpQwHjz2C2-9X9-aMQTfEdZh-5Hmh4c2/view?usp=drive_link

b Available from: https://drive.google.com/file/d/1iY0S5potV4EV6S5egjqVlpkop1Hgwwqp/view?usp=drive_link
